# Impacto de la intervención del laboratorio en la caracterización de la hipervitaminosis B12 en la práctica asistencial

**DOI:** 10.1515/almed-2024-0010

**Published:** 2024-06-03

**Authors:** Sara Fernández-Landázuri, Ramón Baeza-Trinidad, Iván Bernardo González

**Affiliations:** 118003 Servicio de Análisis Clínicos, Hospital Universitario San Pedro, Logroño, Spain; Servicio de Medicina Interna, 118003 Hospital Universitario San Pedro, Logroño, España; Servicio de Análisis Clínicos, 118003 Hospital Universitario San Pedro, Logroño, España

**Keywords:** consultas médicas, costes, hipervitaminosis B12, interferencia, macro-B12

## Abstract

**Objectivos:**

El hallazgo de hipervitaminosis B12 (HB12) no justificado en pacientes asintomáticos desencadena consultas médicas y pruebas diagnósticas, a fin de determinar la etiología. Nuestro objetivo fue probar la eficacia de la intervención del laboratorio en la detección y eliminación de inmunocomplejos con vitamina B12 en la práctica clínica, así como su impacto económico.

**Métodos:**

Es un estudio retrospectivo y longitudinal diseñado para evaluar la estrategia del laboratorio para detectar macrovitamina B12 (macro-B12) en aquellos pacientes con HB12 mayor a 1.000 pg/mL. Se compararon las características clínicas de los pacientes con HB12 derivados a las consultas de Medicina Interna (MI) en el año anterior y posterior a la implantación de la estrategia y se calcularon los costes asistenciales generados en el año de seguimiento de los pacientes.

**Resultados:**

La prevalencia de HB12 en el periodo previo y posterior a la implantación fue del 3,9 % y 3 %, respectivamente. La macro-B12 fue responsable del 25 % de la HB12 iniciales detectadas. El número de pacientes con HB12 derivados a las consultas de MI se redujo en el 41 % tras la implantación, traduciéndose en un ahorro de más de 5.000€.

**Conclusiones:**

La intervención del laboratorio de detección de macro-B12 tiene un claro beneficio asistencial y económico en la práctica clínica.

## Introducción

La determinación de vitamina B12 (VB12), o cobalamina, es una de las más demandadas en la práctica asistencial. Tradicionalmente, su solicitud se ha centrado en la propia deficiencia, en concreto en la anemia megaloblástica y las alteraciones neuropsiquiátricas asociadas a la desmielinización de la médula espinal, nervios periféricos e incluso cerebro [1], [2], [3]. No obstante, cada vez es mayor el interés generado por la hipervitaminosis B12 (HB12), la cual durante mucho tiempo pasó desapercibida e infravalorada [4, 5].

Una dieta equilibrada es suficiente para cubrir los requerimientos nutricionales de VB12, dado que las células humanas no son capaces de sintetizar esta vitamina hidrosoluble. La cobalamina, única vitamina sintetizada exclusivamente por los microorganismos, hace referencia a varias formas de vitamina B12, en la que la adenosilcobalamina y metilcobalamina son las formas biológicas, mientras que las farmacológicas son la cianocobalamina e hidroxicobalamina. Este micronutriente esencial se encuentra principalmente en alimentos de origen animal, entre ellos la carne, lácteos y huevos. También podemos encontrar alimentos enriquecidos con VB12 y un arsenal de suplementos vitamínicos del grupo B administrados vía oral que no requieren prescripción médica. En el tratamiento de la deficiencia de cobalamina puede ser necesario recurrir a la administración parenteral de la misma [2].

La HB12 se denomina a los niveles de VB12 por encima del límite superior de referencia, el cual depende del método analítico empleado. La HB12 se ha asociado a una amplia variedad de entidades clínicas tanto a nivel hepático, renal, autoinmunitario como de neoplasias, principalmente [6]. Los mecanismos moleculares que propician la elevación de VB12 son múltiples, destacando el incremento de proteínas transportadoras en neoplasias, la liberación masiva desde los reservorios hepáticos, la disminución de síntesis de transcobalamina II en patología hepática o el descenso en la filtración de transcobalamina II propia de la patología renal [7]. Son muchos los artículos que enfatizan la relación de la HB12 con las neoplasias, tanto sólidas como hematológicas. De estas últimas, destaca la leucemia mieloide crónica (LMC), policitemia vera (PV) y síndrome hipereosinofílico, introduciéndose incluso la determinación de VB12 como criterio menor y diferenciador de la PV [6, 8], [9], [10]. En cuanto a las neoplasias sólidas, resalta el vínculo entre la HB12 con el cáncer hepático, e incluso asociándose a mayores tasas de mortalidad a corto plazo [11], [12], [13], [14], [15]. A pesar de que la HB12 se ha relacionado tanto con cáncer como con la mortalidad asociada, no hay evidencia suficiente para afirmar la relación causal entre la HB12 y/o el tratamiento con dosis farmacológicas de VB12 con la patología tumoral [14].

Los métodos de análisis de VB12 en la práctica asistencial son los inmunoensayos, en concreto los inmunoquimioluminiscentes. Los cuales no están exentos de interferencias analíticas que conllevan errores en la interpretación de resultados y en el propio manejo de los pacientes [16]. Una de las principales interferencias analíticas descritas en el análisis de VB12 es la formación de inmunocomplejos de inmunoglobulinas IgG o IgM unidas a la cobalamina circulante, denominada comúnmente como macrovitamina B12 (macro-B12). Este inmunocomplejo, funcionalmente inactivo, eleva falsamente la concentración de VB12, llegando incluso a enmascarar la deficiencia vitamínica. La prevalencia de la interferencia por macro-B12 es elevada, suponiendo entre el 10–30 % de las HB12 [5, 17], [18], [19], por lo que es fundamental que los laboratorios la tengan presente y busquen estrategias para identificarlas y eliminarlas en la medida de lo posible [17, 20, 21].

Los laboratorios, cada vez más, están sensibilizados con esta interferencia analítica en el análisis de VB12; sin embargo, faltan proyectos que examinen el papel del laboratorio en este contexto con respecto a su resultado en la clínica.

En el presente trabajo pretendemos evaluar la repercusión clínica y económica que conlleva la estrategia del laboratorio de caracterizar adecuadamente la HB12 tras la eliminación de macro-B12 en aquellos pacientes con HB12 atendidos en las consultas de Medicina Interna (MI) de nuestro centro hospitalario.

## Materiales y métodos

### Diseño del estudio

**Figura 1:**
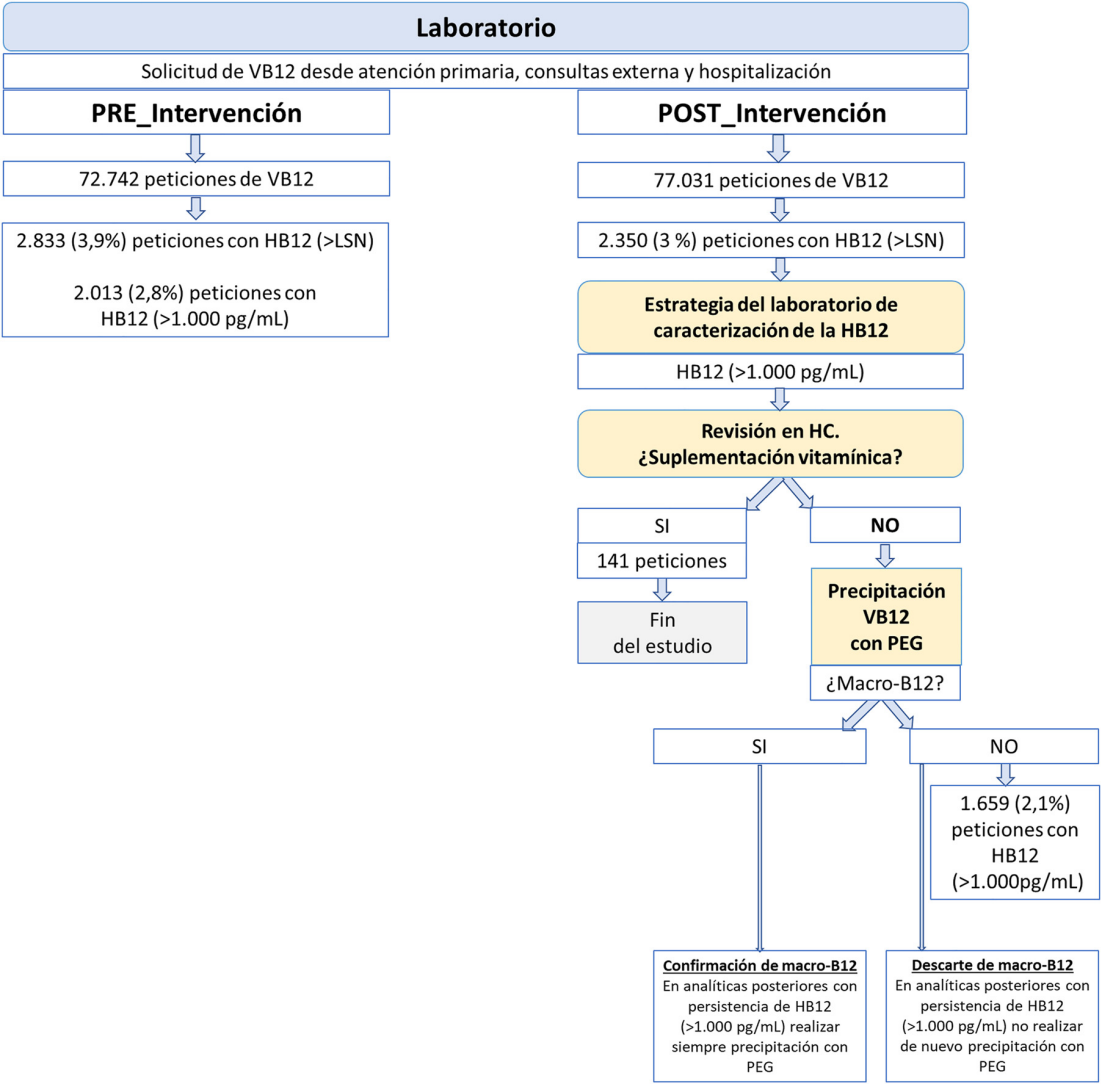
Algoritmo ante elevación de B12. HB12: hipervitaminosis B12; HC: historia clínica LSN: limite superior de normalidad (197 a 771 pg/mL ó 145-569 pmol/L); PEG: polietilenglicol; VB12: vitamina B12.

Se trata de un estudio longitudinal retrospectivo diseñado para evaluar el impacto de la intervención del laboratorio en la detección de macro-B12 en la práctica asistencial y los costes asociados (Figura 1).

El estudio recibió la correspondiente aprobación del Comité de Ética Institucional (Ref CEImLAR P.I. 606).

### Características del laboratorio y centro hospitalario

El laboratorio de Servicio de Análisis Clínicos está adscrito y situado en el Hospital Universitario San Pedro (HUSP. La Rioja, España), el cual asiste a una población de 320.000 habitantes. El laboratorio recibe muestras de pacientes hospitalizados, de consultas externas y atención primaria.

### Abordaje de la hipervitaminosis B12

La estrategia diagnóstica ante paciente con HB12 idiopática derivado a la consulta de MI incluye, junto a la valoración clínica, la solicitud de nueva analítica completa incluidos marcadores tumorales y proteinograma, ecografía abdominal y radiografía de tórax. En los casos necesarios, se completa el estudio con la realización de la tomografía de emisión de positrones (PET-TAC).

### Intervención del laboratorio

En nuestro trabajo, a pesar de que el intervalo de referencia abarca desde 197 a 771 pg/mL, referimos la HB12 a aquella concentración de VB12 superior a 1.000 pg/mL. El laboratorio implantó la estrategia de detección de macro-B12 basada en registrar automáticamente la prueba macro-B12 en el Sistema de Información del Laboratorio (SIL) para aquellos pacientes con un valor de VB12 en suero mayor a 1.000 pg/mL.

La estrategia, incluyó una primera revisión de la prescripción farmacéutica en la historia clínica por parte del responsable de la sección, a fin de descartar aquellos pacientes con suplementación vitamínica de B12.

Una vez excluida la suplementación vitamínica como causa de elevación de VB12, se procedió a valorar la posible interferencia analítica por macro-B12 a través de la precipitación con polietilenglicol (PEG).

En aquellos pacientes en los que se detectó la presencia de macro-B12, si en analíticas posteriores continua con HB12 se realizaba de nuevo la precipitación con PEG. Mientras, en aquellos pacientes en los que se descartó la presencia de macro-B12, si continuaban con HB12 posteriormente, no se repitió el proceso de precipitación.

### Método del laboratorio

La VB12 sérica se cuantificó mediante inmunoensayo de electroquimioluminiscencia (ECLIA) a través del principio de competición en un autoanalizador Cobas 8000 (Roche Diagnostics, Mannheim, Alemania). El intervalo de referencia proporcionado por el fabricante fue 197–771 pg/mL.

El método de detección de inmunocomplejos con VB12 fue la precipitación con PEG. Tras la precipitación, se determinó en el sobrenadante la VB12. Se calculó automáticamente en el SIL el porcentaje de recuperación corregido por la dilución realizada ([concentración de s-B12 tras precipitación con PEG x2/concentración de s-B12 antes de la precipitación con PEG]×100). Se consideró la presencia de macro-B12 cuando el porcentaje de recuperación fue inferior a 60 %, informando el resultado asociado a un comentario indicando el resultado estimado de VB12 por la presencia de macro-B12.

### Pacientes

Se incluyeron en el estudio pacientes con HB12 (>1.000 pg/mL) atendidos en las consultas de MI del HUSP (dado que es la consulta especializada que estudia esta entidad) en el año anterior (pre-intervención) y posterior a la implantación de la estrategia del laboratorio de detección de macro-B12 (post-intervención).

La inclusión en el estudio del paciente fue la fecha de la primera analítica con HB12 (pre o post-intervención). Se realizó el seguimiento de los pacientes durante un año desde su inclusión al estudio. Se excluyeron a los pacientes a los que no se les pudiese hacer el seguimiento en MI y aquellos pacientes que fueron atendidos por este motivo en otros servicios médicos.

Se registraron datos demográficos (edad y sexo), clínicos [presencia de hipertensión, dislipemia, hipotiroidismo, hiperparatiroidismo, anemia, colelitiasis, insuficiencia cardiaca, enfermedad respiratoria crónica, enfermedad renal crónica, hepatopatía, enfermedades autoinmunes, hemocromatosis, cáncer (hepático, mama, colorrectal, gástrico, pancreático y otros), síndrome mieloproliferativo, mielodisplásico, linfoproliferativo y metástasis], farmacológicos (tratamiento con inhibidores de la bomba de protones y la suplementación vitamínica), de morbimortalidad (número de días hospitalizados y mortalidad a un año), analíticos y asistenciales (número de consultas a MI, la solicitud de nueva analítica, ecografía abdominal, radiografía de tórax y otras pruebas de imagen) de los pacientes incluidos en el estudio.

Se calcularon los costes generados por paciente a raíz de los registros de las tarifas de facturación de servicios sanitarios en Rioja Salud. Se calculó el coste de identificación de macro-B12 [Coste identificación paciente con macrovitaminosis-B12 (€)=(Nº test B12 adicionales*Coste test B12)/Nº pacientes identificados]. El coste de la determinación de vitamina B12 en nuestro centro fue de 1,29 €. Se desestimó los costes derivados de PEG, fungibles y personal, así como otros costes indirectos e intangibles.

### Análisis estadístico

El análisis estadístico de los resultados se realizó con el programa *Statistical Package for the Social Sciences 22.0* (SPSS Inc., Chicago, IL, EE.UU).

La distribución no gaussiana de los datos se comprobó con el test de normalidad de Kolmogorov–Smirnov. Las variables continuas se expresan como medias±DE o mediana y rango intercuartílico (según normalidad), mientras que los datos categóricos se presentan en valores absolutos (n) y porcentuales (%).

Se empleo la prueba de *Chi* cuadrado para investigar diferencias en las variables categóricas entre los grupos. La prueba t de Student o la prueba U de Mann Whitney se emplearon para comparar las muestras no pareadas, en su caso. Un p bilateral <0,05 se consideró estadísticamente significativo.

## Resultados

En el periodo estudiado se observa un incremento del 5,9 % del nivel asistencial en el laboratorio en función del número de peticiones solicitadas de VB12, así como un incremento del 4,6 % de la actividad asistencial según el número de consultas totales del Departamento de Medicina Interna de nuestro centro (Figura 1).

En nuestra área de salud la HB12, niveles superiores a 1.000 pg/mL, representó el 3,9 % y 3 % en el periodo pre y post-intervención, respectivamente.

En el periodo post-intervención se constató que el 6 % de las elevaciones de B12 iniciales se debieron a la suplementación vitamínica con B12 y se detectó un 24,9 % de macro-B12. De forma colateral al objetivo del presente estudio, se detectaron a 10 pacientes con deficiencia de VB12 (<200 pg/mL) mediante la precipitación con PEG en los cuales su valor inicial se enmarcó dentro de la HB12.

El número de pacientes con HB12 atendidos en las consultas de MI se redujo considerablemente al comparar ambos periodos. En el periodo pre-intervención se atendieron a 61 pacientes con HB12 (7,2 % del total de las consultas de MI) y en el post-intervención a 36 pacientes (4 %), lo cual supuso una reducción del 41 %. La población atendida en las consultas de MI en ambos periodos fue similar (Tabla 1). En cuanto a las variables asociadas a patología no se observaron diferencias significativas, a excepción de la presencia de diabetes mellitus (pre-intervención 22 % post-intervención 6 %; p: 0,030) y anemia (pre-intervención 11 % y post-intervención 0 %; p: 0,034). Como se ha mencionado, la estrategia diagnóstica implantada en el nuestro centro ante un paciente con HB12 sin causa justificada genera una serie de consultas y pruebas complementarias adicionales, destacando un descenso en el número de analíticas solicitadas y un aumento del número de pruebas de imagen (Tabla 2).

**Tabla 1:** Características clínicas y de morbimortalidad de los pacientes con elevación de vitamina B12 atendidos en las consultas de Medicina Interna.

Características	Pre-intervención (61 pacientes)	Post-intervención (36 pacientes)	Valor p
Edad, años (media, DE)	68±14	68±17	0,692
Sexo, n; % mujeres	39; 64 %	21; 58 %	0,368
Raza, n; % Caucásica	58; 95 %	33, 92 %	0,160
Tumor sólido, n; %	11; 18 %	3; 8,3 %	0,155
Tumor hematológico, n; %	5; 8,2 %	7; 19,4 %	0,970
Tumor sólido y hematológico, n; %	16; 22,2 %	10; 27,8 %	0,454
Metástasis, n; %	2; 3,3 %	3; 8,3 %	0,454
Mortalidad a 1 año, n; %	11; 18 %	10; 27,8 %	0,191

DE, desviación estándar, %, porcentaje.

**Tabla 2:** Solicitud de pruebas analíticas y de imagen solicitadas a los pacientes con elevación de vitamina B12 atendidos en las consultas de Medicina Interna.

Pruebas complementarias	Pre-intervención (61 pacientes)	Post-intervención (36 pacientes)	Valor p
Solicitud de nueva analítica tras elevación de vitamina B12, n; %	48; 78,3	23; 63,9	0,970
Solicitud de ecografía abdominal tras elevación de vitamina B12, n; %	14; 23,3	4; 11,1	0,110
Solicitud de radiografía tórax tras elevación de vitamina B12, n; %	12; 20	6; 16,7	0,452
Solicitud de PET-TAC tras elevación de vitamina B12, n; %	1; 1,7	36; 8,3	0,147

Los pacientes con HB12 atendidos en las consultas de MI generaron una serie de costes derivados de la propia asistencia y de pruebas complementarias que fueron destacables (Tabla 3). Se apreció a nivel global una reducción de gasto en todos los costes en el periodo post-intervención, destacando una disminución del coste de las pruebas de imagen, a excepción de los derivados del PET-TAC (Tabla 3). Teniendo en cuenta que la estrategia de caracterización de VB12 mediante la precipitación con PEG requirió una inversión por parte del laboratorio de 2.433 € y que la diferencia del coste total de los pacientes atendidos en las consultas de MI entre ambos periodos fue de 7.528 €, consiguió la reducción total de gasto de 5.095 €. En cuanto a la normalización del coste por paciente es mayor en el periodo post-intervención en los derivados de la consulta médica y de las pruebas de imagen (Tabla 3).

**Tabla 3:** Coste total y normalizado por paciente de la atención médica de los pacientes con elevación de vitamina B12 atendidos en las consultas de Medicina Interna.

Categoría del coste	Pre-intervención (61 pacientes)	Post-intervención (36 pacientes)	Diferencia del gasto	Coste/paciente pre-intervención	Coste/paciente post-intervención	Diferencia del gasto/paciente
Consulta MI, €	22.350	14.031	−8.319	366,4	389,8	+23,4
Analítica, €	1.537	782	−755	25,2	21,7	−3,5
Ecografía abdominal, €	826	236	−590	13,5	6,6	−6,9
Radiografía de tórax, €	240	120	−120	3,9	2,2	−0,6
PET-TAC, €	1,128	3.384	+2.256	18,5	94,0	+75,5
Coste asistencial total	26.081	18.553	−7.528	427,6	515,4	+87,8
Coste del laboratorio (caracterización de HB12)	0	2.433	+2.433			
Coste total estrategia en MI			−5.095			

MI, medicina interna; HB12, hipervitaminosis B12; €, Euros.

## Discusión

La HB12 se ha relacionado con entidades clínicas muy diversas, entre las que resalta principalmente la patología hepática, neoplasias y enfermedades autoinmunes, e incluso como factor pronóstico de estas enfermedades [6, 11, 12, 15]. Este hecho hace que la detección fortuita de HB12, en concreto en pacientes asintomáticos y sin causa que la justifique, desencadene una serie de actuaciones clínicas basadas en consultas clínicas y solicitud de pruebas diagnósticas complementarias para determinar su origen, buscando enfermedades ocultas en el paciente [4, 7].

En este contexto, se estima que en torno al 60–70 % de las decisiones médicas están basadas en los resultados analíticos, por lo que el laboratorio tiene la responsabilidad de proporcionar datos fiables, evitando generar falsos resultados que conlleven a catalogar erróneamente a sujetos sanos como enfermos, con las correspondientes implicaciones personales, laborales y económicas [22], [23], [24], [25]. En este sentido, la innovación tecnológica, la estandarización de los métodos de análisis, el aseguramiento de un sistema de calidad y los avances informáticos han permitido reducir considerablemente los errores del laboratorio [24].

No obstante, los métodos empleados en los laboratorios clínicos para el análisis de VB12 se basan en inmunoensayos, los cuales no están libres de interferencias analíticas [16]. La formación de inmunocomplejos constituye una fuente importante de interferencias, debida a la capacidad que presenta el anticuerpo de unirse *in vivo* al analito modificando no sólo sus características, sino conservando la capacidad de reaccionar con los inmunoensayos, provocando falsas elevaciones. Esta interferencia ha sido descrita en numerosos estudios, asociándose principalmente a la prolactina, de ahí que los laboratorios tengan implantados procedimientos para la detección de la macroprolactina y su eliminación basada en la precipitación con PEG [25], [26], [27]. Esta interferencia también se ha descrito en otras hormonas como la TSH, FSH e incluso enzimas como la creatin-Kinasa [16, 28, 29]. En los últimos años, ha crecido el interés en los laboratorios por detectar este tipo de interferencias analíticas en el estudio de la VB12, en especial en aquellos pacientes con HB12 asintomática e inexplicable. La técnica de referencia para la detección de inmunocomplejos es la cromatografía de exclusión molecular; sin embargo, la complejidad intrínseca de la misma y los tiempos de respuesta invalidan su utilidad a nivel asistencial, al igual que con la cromatografía de filtración en gel [17, 30]. El laboratorio clínico necesita implantar técnicas y protocolos viables, por ello la precipitación con PEG es el método de detección de macro-B12 en la práctica diaria. En disolución acuosa, este polímero no iónico es capaz de precipitar proteínas de alto peso molecular, tales como los inmunocomplejos, de una forma sencilla, rápida y económica. A pesar del perjuicio derivado de un posible arrastre inespecífico de otras moléculas de pequeño tamaño como la cobalamina, consideramos muy útil y práctico este método de detección de macro-B12 para la caracterización adecuada de HB12. De hecho, la prevalencia de macro-B12 en nuestra población es del 24,9 %, datos similares a los publicados en otras cohortes de pacientes [17, 20, 21]. Estas cifras justifican la necesidad de instaurar protocolos para abordar esta interferencia analítica, con un enfoque similar al logrado con la macroprolactina [27]. Por esta razón, y a pesar de las posibles limitaciones del método, consideramos de utilidad indicar en el informe la concentración de VB12 tras precipitación con PEG y corrección por el factor de dilución.

En líneas generales, la causa más frecuente de HB12 se ha achacado al tratamiento con suplementos de hidroxicobalamina o cianocobalamina vía oral o intramuscular [2, 7], aunque en nuestra población es llamativo que la prevalencia de suplementación es menor incluso que la detección de macro-B12, si bien es cierto que gran parte de los suplementos vitamínicos administrados por vía oral no constan en las historias clínicas debido a que no requieren prescripción farmacológica.

Una forma indirecta de evaluar el beneficio de la estrategia del laboratorio se plasma en la reducción de HB12 pre y post intervención a pesar del incremento del 5,9 % de la carga asistencial. Estos datos son extrapolables a nivel clínico, donde se ha conseguido una reducción del número de pacientes con HB12 derivados a las consultas de MI aun cuando el número total de consultas aumentó. Un aspecto destacable es que gracias al método de detección de macro-B12 se desenmascararon a 10 pacientes con deficiencia de VB12, dos de los cuales habían sido previamente remitidos a la consulta de MI para proceder al estudio de HB12 asintomática y no justificada.

Las características de la población estudiada en ambos periodos fueron semejantes, excepto en la mayor prevalencia de diabetes mellitus y anemia en el grupo pre-intervención, donde los autores no encontraron explicación razonable a esta circunstancia. Un reciente estudio demostró que tanto la deficiencia como la elevación de VB12 está asociado significativamente con un mayor riesgo de mortalidad cardiovascular en diabéticos tipo 2 [31]; sin embargo, en nuestra población no se pudo constatar dicha asociación.

En la cohorte estudiada, los resultados obtenidos apuntan que el grupo de post-intervención presentó mayor morbilidad atendiendo a mayores tasas de neoplasias a nivel global y metástasis, así como mortalidad a un año. Esta mayor complejidad de paciente en el grupo post-intervención se reflejó en un mayor coste por paciente tanto a nivel de consultas médicas como de pruebas de imagen. Esto pone de manifiesto que la intervención del laboratorio permitió caracterizar mejor a los pacientes, evitando crear “falsas HB12” que generan consultas y costes innecesarios. En este sentido podemos suponer que el grupo pre-intervención pudo estar formado por algunos pacientes con HB12 cuyos valores se debían a la macro-B12 y como consecuencia la carga de patología en dicho grupo pudo ser menor.

Son cada vez más frecuentes los trabajos donde se reporta la interferencia por inmunocomplejos; sin embargo, escasean los estudios que valoran el impacto del ejercicio del laboratorio en la práctica asistencial, y mucho menos su repercusión económica [5, 17]. Por lo que el propósito principal de este estudio radicó en evaluar el efecto de una actuación del laboratorio en la clínica. Este es un enfoque similar a los planteados por otros autores que enmarcan los nuevos modelos del laboratorio centrados de forma activa en la toma de decisiones [32], [33], [34], [35]. Los resultados demuestran que la estrategia del laboratorio fue coste-efectiva. La inversión inicial del laboratorio en la identificación y eliminación de la interferencia por inmunocomplejos fue de 2.433 €, cifra tres veces inferior a la diferencia de costes asistenciales generados entre el periodo pre y post-intervención en una única área, las consultas de MI. Por lo que es presuponer el ahorro es aún superior si se extrapola a todos los pacientes.

Una de las limitaciones del estudio fue calcular el impacto de la estrategia del laboratorio en las consultas de MI, excluyendo a las HB12 atendidas en atención primaria (si bien es cierto, que en general en nuestra área la gran parte de las HB12 no justificadas son derivadas a MI). Adicionalmente, cabe señalar que existe un pequeño porcentaje de pacientes donde, aun descartándose la macro-B12, este aún presenta HB12 sin causa justificable. Y es en estas situaciones, en las que la clínica y la analítica no van de la mano, donde cobra relevancia la interacción entre el laboratorio y los clínicos con objeto de encontrar soluciones a las discrepancias detectadas.

En conclusión, es fundamental que el laboratorio ejerza un rol activo en la caracterización adecuada de las HB12, enfatizando en la detección y eliminación de inmunocomplejos que artefactan el resultado, contribuyendo a una disminución de las pruebas diagnósticas, una optimización de la toma de decisiones y una reducción de costes. Este trabajo pone de manifiesto las consecuencias del papel del laboratorio en la clínica y la economía de salud.
